# A quantitative PCR to detect non-toxigenic *Clostridioides difficile*

**DOI:** 10.1128/spectrum.01608-24

**Published:** 2024-12-11

**Authors:** Khurshida Begum, Hubert C. Chua, M. Jahangir Alam, Kevin W. Garey, Jinhee Jo

**Affiliations:** 1 Department of Pharmacy Practice and Translational Research, University of Houston College of Pharmacy, Houston, Texas, USA; Johns Hopkins University, Baltimore, Maryland, USA

**Keywords:** *Clostridioides difficile*, non-toxigenic, diagnostics, stewardship

## Abstract

*Clostridioides difficile* species lacking toxin genes (non-toxigenic *C. difficile* or NTCD) may confer protection against CDI. However, current diagnostic tests detect either toxin proteins or toxin genes and cannot detect NTCD. This study developed a molecular testing method that uniquely identified NTCD and assessed its prevalence in a clinical cohort. A quantitative PCR (qPCR) assay was developed and validated using reference strains. Analytic sensitivity was determined using DNA from reference NTCD strains, and qPCR efficiency was assessed using the slope of the standard curves of DNA dilutions. A random selection of 95 clinical stool samples, tested using the GDH enzyme and toxin enzyme immunoassay (EIA), was used to evaluate the prevalence of NTCD in hospitalized patients tested for CDI. The KB-1/KB-2 primers and probe designed were specific for NTCD strains and did not amplify with toxigenic *C. difficile* or other related strains. The NTCD qPCR assay analytical sensitivity was linear between 3 × 10^1^ and 3 × 10^6^ gDNA (*R*^2^ = 0.999; *P* < 0.0001). No NTCD was found in 25 GDH-EIA –/– samples compared to 5 of 25 (20%) GDH-EIA +/– samples and 2 of 23 (8.7%) GDH-EIA +/+ samples. Of samples detected with NTCD, median NTCD DNA was 33,039 (IQR: 22.449–45.688) in GDH-EIA +/– samples and 370 [IQR: 159–583] in GDH-EIA +/+ samples. The new qPCR NTCD assay identified NTCD colonization in 7% of hospitalized patients tested for CDI. This NTCD assay may have important implications for diagnostic and antimicrobial stewardship as colonization with NTCD strains may offer protection against CDI.

**IMPORTANCE:**

Current diagnostic strategies do not detect non-toxigenic *Clostridioides difficile* (NTCD) strains, which may provide protection against *C. difficile* infection (CDI). Detecting these strains is critical as it underscores the importance of avoiding unnecessary antibiotic treatment in patients colonized with NTCD. To better guide clinical decisions and enhance the understanding of NTCD epidemiology, molecular assays that specifically target non-coding regions unique to NTCD strains are needed. In this study, we developed and validated a qPCR assay capable of uniquely identifying NTCD strains. This innovative assay holds significant potential for applications in public health, infection control, diagnostic, and therapeutic strategies related to CDI.

## INTRODUCTION

*Clostridioides difficile* is a Gram-positive, spore-forming, toxin-producing obligate anaerobe responsible for *C. difficile* infection (CDI) accounting for over 450,000 cases and 13,000 deaths annually in the USA (1, 2). CDI is mediated via the production of two large exotoxins, toxin A and toxin B, encoded by genes *tcdA* and *tcdB*, respectively (3). Toxin production results in the disruption of tight junction between colonic epithelial cells, loss of epithelial integrity, and onset of diarrhea. The toxin genes are located within a 19.6 kb region of the chromosome known as the pathogenicity locus, or PaLoc.(4) Non-toxigenic *C. difficile* (NTCD), which lacks the PaLoc, frequently colonizes hospitalized patients. In NTCD strains, the entire PaLoc is usually replaced by a highly conserved 75 or 115 bp non-coding region at the insertion sequences or a truncated, non-functional PaLoc can also be present (4, 5). These strains may confer a benefit to prevent colonization and infection with toxigenic *C. difficile*. For example, in a phase 2 clinical trial, treatment with a NTCD strain 10^4^ spores/day for 7 days decreased the incidence of recurrent CDI (6).

Current CDI diagnostic tests detect one or both *C. difficile* toxin proteins by enzyme immunoassay (EIA) or toxin genes by nucleic acid amplification test (NAAT). Each diagnostic strategy has unique characteristics. NAAT-based diagnostics can detect asymptomatic colonization, leading to false positives. EIA-based diagnostics lack sensitivity, leading to a higher likelihood for false negatives (7). To increase testing sensitivity, a two-step diagnostic algorithm for CDI using a combination of toxin EIA and detection of a highly conserved glutamate dehydrogenase (GDH) enzyme has been widely adopted (8). Biologically, GDH is present in all *C. difficile* strains whether toxigenic *C. difficile* or NTCD (9). Although sensitive, GDH tests lack specificity as they cannot distinguish between toxigenic and non-toxigenic strains. Up to 30% of samples using the two-step testing can yield indeterminate results (i.e., GDH+/Toxin–) which may be due to the presence of NTCD strain or a false negative with a toxigenic *C. difficile* strain. These indeterminant tests are frequently followed by NAAT to check for false negative EIA results and possible start of antibiotics. However, NAAT detection of colonization is still possible. Indeterminate results caused by colonization with NTCD strains may be clinically relevant as they potentially provide protection against CDI (6, 10, 11) and emphasize the importance of not treating these patients with antibiotics. However, molecular assays targeting the non-coding regions unique to NTCD strains to help guide these clinical decisions or study NTCD epidemiology have not been developed. Thus, this study aimed to develop a molecular testing method to uniquely identify and quantify NTCD direct from stool.

## MATERIALS AND METHODS

### Samples and isolates

Leftover stool samples from patients tested for possible CDI were collected as a part of a multicenter cohort study and brought to a centralized research laboratory at the University of Houston College of Pharmacy for further testing (12). The cohort in this analysis included adult patients admitted to a university-affiliated quaternary care hospital or their respective associated community hospitals in the greater Houston area between 2022 and 2023. Patients were included in the study if they were tested for CDI as part of routine clinical care, using the two-step, GDH-EIA diagnostic (*C. DIFF QUIK CHEK COMPLETE*, Techlab, Blacksburg, VA). Results for the GDH-EIA were categorized as positive (+/+), negative (–/–), or indeterminate (+/–). One hundred stool samples were obtained and stored at −80°C until analysis. This study was approved by the Committee for the Protection of Research Subjects at the University of Houston (CPHS 000128) and associated hospitals.

### DNA extraction

Stool DNA was extracted using the DNeasy PowerSoil Pro Kit (Qiagen, Cat# 47016) in a QiaCube automated DNA extraction system (Qiagen, Hilden, Germany) according to manufacturer instructions. Briefly, 250 mg stool was transferred into a PowerBead Pro Tube along with 200 µg RNase A and 800 µL of CD1 solution. The tubes were vortexed briefly and transferred into an adapter and vortexed at maximum speed for 2 min. Thereafter, tubes were centrifuged at 15,000 × *g* for 1 min, and about 600 µL supernatant was used for DNA extraction. The DNA was eluted in 70 µL elution solution C6 and stored at −80°C until analysis.

### PCR assay development

**Fig 1:**
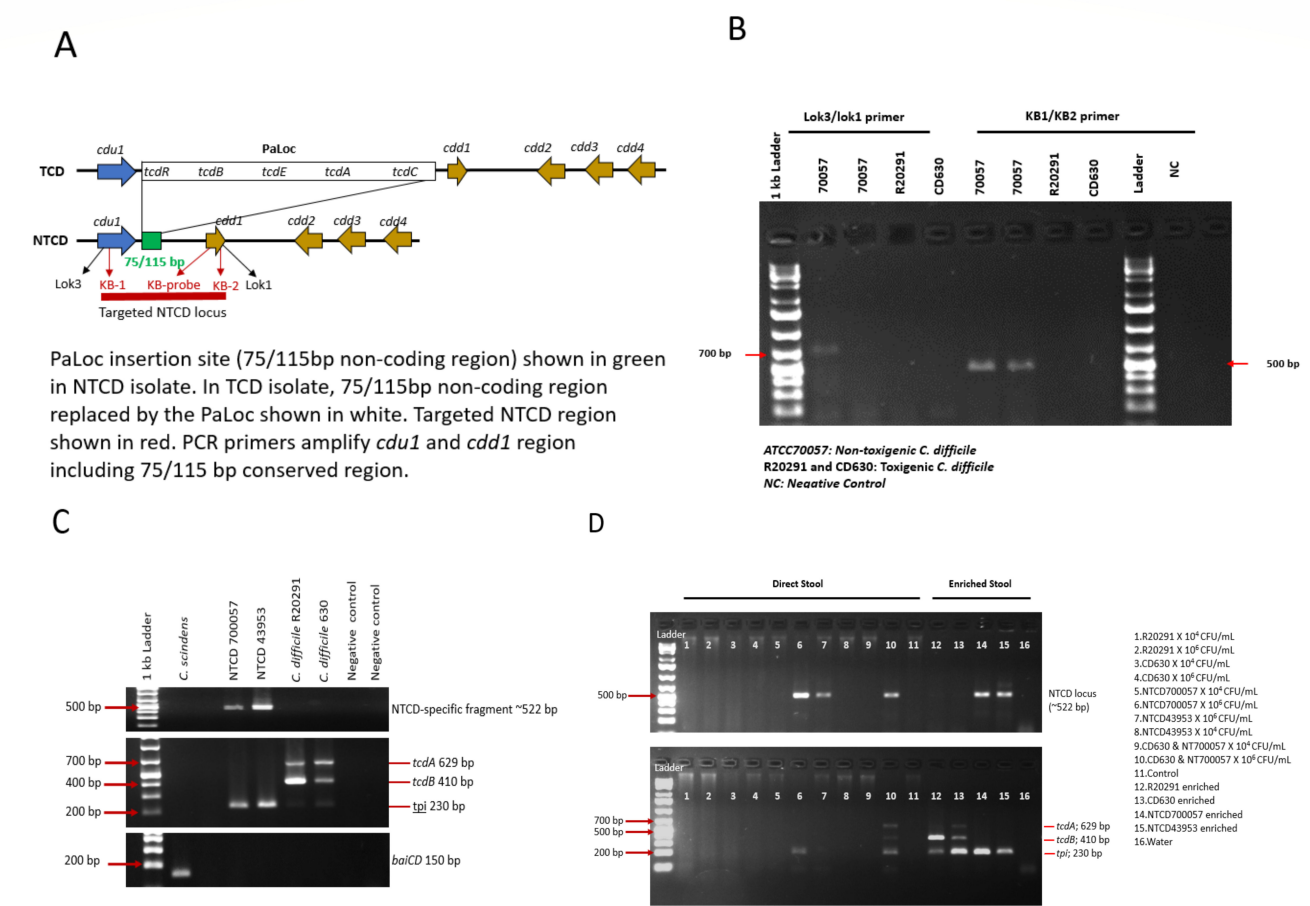
Schematic drawing of PaLoc and NTCD-specific fragment regions in TCD and NTCD isolates and development of NTCD PCR.

Our existing multiplex polymerase chain reaction (PCR) assay for toxigenic *C. difficile* includes *tpi* (universal gene present in all *C. difficile* strains) and the genes encoding for toxin A (*tcdA*) and toxin B (*tcdB*) (13, 14). To detect NTCD strains, we previously used time-intensive anaerobic growth culture followed by PCR confirmation, with the absence of toxin genes. Our PCR assay was modified with new primers (forward primer, KB-1, 5′ CGAAGAGGAGCTAACAGAGGAA 3′ and reverse primer KB-2, 5′ TACATTTGTGCTGGGTTTGC 3′) to target the highly conserved, 115 bp non-coding region unique to NTCD strains and compared against previously published primers (Lok3.lok1 primers; Table 1) (4). The newly designed primer location for PCR is illustrated in Fig. 1A which shows hybridization and amplification of partial *cdu1* and *cdd1* regions, including the highly conserved 75/115 bp regions. Primer sequences were evaluated using the Basic Local Alignment Search tool (BLAST) to assess the specificity of the primers toward the 115 bp non-coding region. All primers were ordered from ThermoFisher Scientific (Hampshire, England) as custom standard DNA oligos. The PCR assay was performed per manufacturer’s protocol, and the gel was stained with ethidium bromide and photographed under UV light.

**TABLE 1:** Primers and probes used in this study

Primers	Sequence (5′−3′)	Gene	Reference
tpi-F tpi-R	AAAGAAGCTACTAAGGGTACAAA CATAATATTGGGTCTATTCCTAC	*tpi*	(15)
tcdA-F3345 tcdA-R3969	GCATGATAAGGCAACTTCAGTGGTA AGTTCCTCCTGCTCCATCAAATG	*tcdA*	(16)
tcdB-F5670 tcdB-R6079A tcdB-R6079B	CCAAARTGGAGTGTTACAAACAGGTG GCATTTCTCCATTCTCAGCAAAGTA GCATTTCTCCGTTTTCAGCAAAGTA	*tcdB*	(16)
Lok3 Lok1	TTTACCAGAAAAAGTAGCTTTAA AAAATATACTGCACATCTGTATA	NTCD-specific fragment	(4)
KB-1 KB-2 KB-probe	CGAAGAGGAGCTAACAGAGGAA TACATTTGTGCTGGGTTTGC FAM- AAATTCCAGGGGTAGGGAAA	NTCD-specific fragment	Current study
baiCD-F baiCD-R	CAGCCCRCAGATGTTCTTTG GCATGGAATTC HACTGCRTC	*baiCD*	(17)
Sg-clept-F Sg-clept-R	GCACAAGCAGTGGAGT CTTCCTCCGTTTGTCAA	16s rRNA	(18)

### NTCD qPCR assay development

The NTCD qPCR assay was developed by incorporating the NTCD sequencing region into the current qPCR (19). The standard PCR KB-1 forward primer (5′ CGAAGAGGAGCTAACAGAGGAA 3′) and KB-2 reverse primer (5′ TACATTTGTGCTGGGTTTGC 3′) described above were used along with a FAM-tagged probe (KB-probe, FAM AAATTCCAGGGGTAGGGAAA, TaqMan QSY probe, ThermoFisher Scientific) (Table 1). Using the QuantStudio5 Real-Time PCR System (Thermo Scientific, Hampshire, England), qPCR was performed on each sample in triplicate at a final volume of 20 µL containing 25 ng DNA template, each primer at 0.5 µM (forward primer KB1 and reverse primer KB2), and a FAM-tagged probe (KB-probe) at 0.25 µM using TaqPath ProAmp Master Mix (Applied Biosystems, Cat #A30866). Cycle conditions for the qPCR were one cycle of initial denaturation at 95°C for 10 min followed by 40 cycles of 95°C for 15 s and 60°C for 1 min with auto ramp rate and auto increment.

### Determination of sensitivity and specificity of the PCR and qPCR

To determine sensitivity and specificity, genomic DNA (gDNA) was extracted from cultured NTCD reference strains (NT700057 and NT43953), toxigenic *C. difficile* strains (R20291 and CD630), and related Clostridial strain (*Clostridium scindens*). DNA extraction of bacterial species was performed using the AnaPrep Bacterial DNA Extraction Kit (Cat#Z1322006) in an automated DNA extraction system (AnaPrep 12, BioChain Institute Inc., Newark, CA, USA). The quantity and quality of gDNA were measured using a QuickDrop (ThermoFisher Scientific, Hampshire, England), absorbance ratio at A260/A280, and 1% agarose gel electrophoresis. NTCD PCR products for relevant genes were assessed on agarose gels on separate PCR runs for the NTCD-specific fragment, toxin genes (*tcdA* and *tcdB*), and *tpi*, bile acid-inducible gene (*baiCD* present in *C. scindens*) (17). Sanger sequencing was performed to confirm primer-specific NTCD-specific fragment amplification.

Analytic sensitivity of NTCD qPCR assay was determined using DNA from referenced NTCD strains (concentrations: 10^1^ to 10^8^ copy number). Standards were prepared by performing PCR using species-specific primers on the two reference NTCD strains. A range of 10-fold serially diluted gDNA standard was prepared on each qPCR plate and ran in triplicate. qPCR efficiency was determined using the slope of the standard curves of DNA dilutions. The cycle of quantification (*C*_q_) value for each dilution was recorded, and the lowest concentration of DNA obtained from the analytic sensitivity experiments was defined as the lowest level of genomic analytic sensitivity. Threshold cycle values were converted to copies per nanogram of DNA using a standard curve. *R*^2^ values of standards curves were calculated, and copies per sample were calculated accounting for the initial DNA concentrations in the sample. Toxigenic *C. difficile* and related *Clostridium* strains were used to determine analytical specificity.

Three *C. difficile* negative stool samples (GDH–/Toxin–/PCR–/*C. difficile* growth negative) were pooled to create a homogenized mass and aliquoted to 500 mg per tube. Each tube was spiked with different *C. difficile* strains at 10^4-8^ concentrations. Bacterial suspensions with vegetative cells at 10^4-8^ colony-forming units/mL (CFU/mL) were prepared for NTCD strains (NT700057 and NT43953), toxigenic *C. difficile* strains (R20291 and CD630), and a combination toxigenic and non-toxigenic strains (CD630 and NT700057). Suspension aliquots (0.1 mL) were transferred into 0.9 mL *C*. *difficile* negative stool. DNA was extracted from the stool sample within an hour of spiking to determine the analytic sensitivity of the qPCR directly from stool. A negative control was included for each run. qPCR was also performed on DNA isolated directly from each *C. difficile* suspension and after a stool enrichment step allowing for *C. difficile* to grow anaerobically at 37°C for 24 h before testing.

### NTCD detection in clinical samples tested by the two-step, GDH-EIA diagnostic

Clinical stool samples tested for CDI using the GDH-EIA two-step diagnostic were tested for NTCD using the qPCR. The overall proportion of NTCD positive and NTCD DNA quantity based on GDH-EIA results was calculated.

Data will be made publicly available upon request.

## RESULTS

### Standard PCR development

The KB-1/KB-2 primers designed (~522 bp) for this study were compared against the previously published Lok3/lok1 primers (700 bp) (Table 1). Primer specificity was confirmed by Sanger sequencing and NCBI BLAST. Both primers set amplified conserved *cdu1* and *cdd1* regions (Supplementary document). Both primers were specific for NTCD strains and did not amplify with toxigenic *C. difficile* strains R20291 or CD630 or with other related *C. difficile* strains. However, the KB-1/KB-2 primers for PCR amplified the two tested NTCD strains in all experiments, while the Lok3/lok1 primers amplified only one of the two tested NTCD strains (Fig. 1B). Based on these results, the KB-1/KB-2 primers were used for all further experiments.

The standard PCR assay was developed using NTCD reference strains (NTCD 700057 and NTCD 43953), toxigenic *C. difficile* strains (R20291 and CD630), and Clostridial strain (*C. scindens*) (Fig. 1C). The NTCD-specific fragment (~522 bp) was accurately detected in NTCD reference strains while remaining undetected in toxigenic or related strains on the gel. In spiking experiments using different concentrations (10^4^–10^6^ CFU/mL), the NTCD-specific fragment was detected in all NTCD strains and remained undetected in toxigenic *C. difficile* strains in samples that underwent a stool enrichment step. However, it was detected in only three of six NTCD strains in samples that were tested immediately following DNA extraction without an enrichment step (Fig. 1D).

### qPCR development

**Fig 2:**
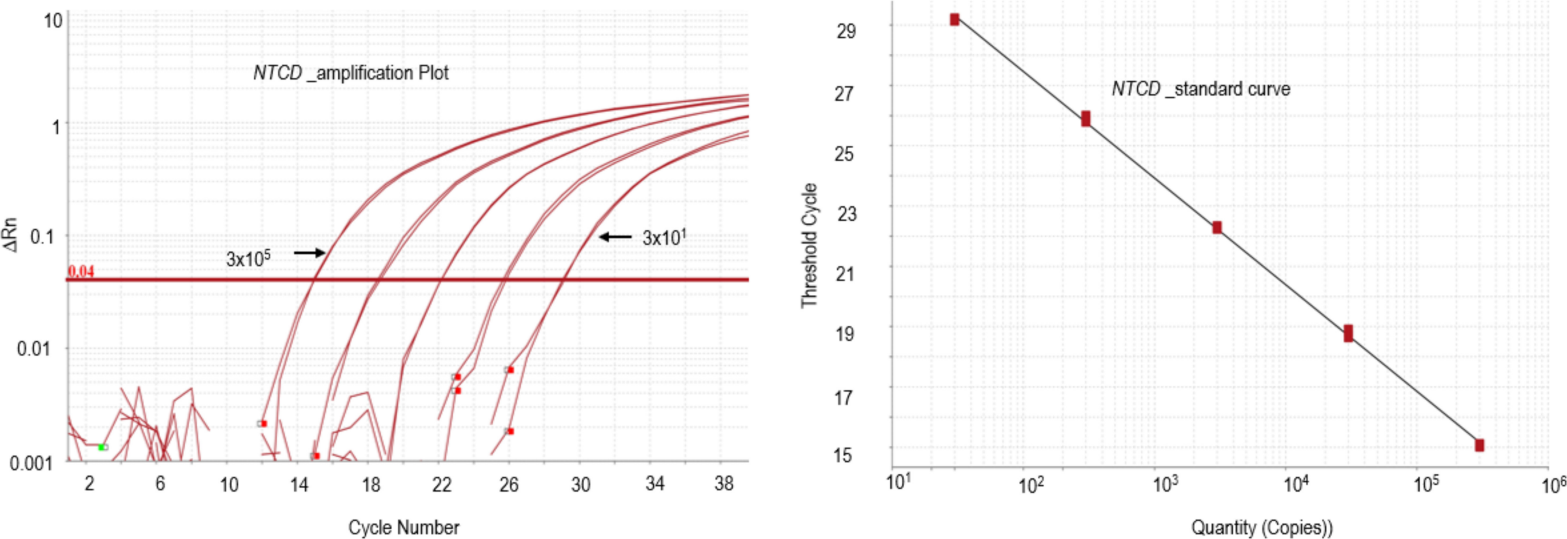
Amplification plots and standard curves for non-toxigenic *C. difficile* (NTCD). Standard Curves were generated with five 10-fold dilutions ranging from 3 × 10^1^ to 3 × 10^6^ genomic DNA.

The NTCD qPCR assay analytical sensitivity (Fig. 2) was linear between 3 × 10^1^ and 3 × 10^6^ gDNA (*R*^2^ = 0.999; *P* < 0.0001). The NTCD qPCR yielded negative results with spiking studies using toxigenic *C. difficile* (R20291 and CD630) and positive results with NTCD (NT43953 and NT700057) and in toxigenic and non-toxigenic spiking experiments using CD630 and NT700057 together (Table 2).

**TABLE 2:** NTCD DNA quantity results of spiking experiments in NTCD and related species^*a*^

		NTCD quantity (Mean ± SE)		
	CFU log spike	1 × 10^4^	1 × 10^6^	1 × 10^8^
Strain	R20291	BLQ	BLQ	BLQ
	CD630	BLQ	BLQ	BLQ
	CD630_NT700057	140 ± 30	10,169 ± 881	NA
	NT43953	423 ± 52	44,941 ± 9,106	11,046 ± 1,131
	NT700057	291 ± 44	41,094 ± 3,150	5,723 ± 475

^*a*^ NTCD, non-toxigenic *C. difficile*; BLQ: below the limit of quantification; CFU: colony-forming units. Note: R20291 and CD630 are toxigenic *C. difficile* strains, NT700057, NT43953, and NT700057 are NTCD; NA, not applicable.

### Evaluation of NTCD-specific PCR on clinical samples

Ninety-five clinical stool samples tested for CDI using the GDH-EIA two-step diagnostic were tested for NTCD using the qPCR (GDH-EIA –/–: *n* = 47; +/–: *n* = 25; and +/+: *n* = 23). No NTCD was identified in 25 GDH-EIA –/– samples compared to 5 of 25 (20%) GDH-EIA +/– samples and 2 of 23 (8.7%) GDH-EIA +/+ samples. Of samples detected with NTCD, median NTCD DNA was 33,039 (IQR: 22.449–45.688) for GDH-EIA +/–samples and 370 [IQR: 159–583] for GDH-EIA +/+ samples.

## DISCUSSION

Clinical manifestations of CDI can range from mild diarrhea to toxic megacolon and death (20). Toxigenic *C. difficile* is identified by the presence of toxin proteins via EIA or toxin genes by NAAT. NTCD strains exist in which the PaLoc is absent and is generally replaced by a non-coding 115/75 bp region (21). NTCD strains have been shown to be protective against CDI as observed in a hamster model of the disease (22, 23) and human clinical trials (6, 11). NTCD is also relevant in CDI diagnostics using the GDH-EIA two-step diagnostic in which discordant results (GDH-EIA +/–) may be due either to lack of EIA sensitivity to *C. difficile* toxins or the presence of a NTCD strain. Given these therapeutic and diagnostic implications, the goal of this study was to develop a qPCR capable of detecting NTCD in clinical stool samples. Using NTCD and comparator reference standard strains as well as clinical stool samples from hospitalized patients tested for CDI using the GDH-EIA two-step diagnostic, we developed an analytically sensitive method that was able to detect and quantitate NTCD DNA in stool samples. To the best of our knowledge, this is the first developed qPCR targeting detection of NTCD strains.

The NTCD qPCR demonstrated analytical sensitivity as low as 3 × 10^1^ NTCD gDNA and was able to detect NTCD in spiking studies of NTCD strains as low as 1 × 10^4^ CFU/mL. Using this assay, we determined that 20% of clinical stool samples with discordant GDH-EIA two-step diagnostic results harbored NTCD DNA. Nine percent of positive GDH-EIA tests harbored NTCD DNA, but the concentrations of DNA were relatively lower than those observed in the NTCD-positive samples with the discordant GDH-EIA results. Mixed *C. difficile* strain infection has been reported in approximately 7%–16% of all cases (24). In this study, we similarly observed that 6% of our samples had both NTCD and toxigenic *C. difficile*. The clinical significance of mixed *C. difficile* strain infection including NTCD detection remains unknown and requires further evaluation. Previous studies have similarly reported the prevalence of NTCD in adults with symptomatic diarrhea to range between 3% and 26%, while in asymptomatic hospitalized adults, the range increased up to 46% (25). Studies conducted outside of the USA have also reported that 12%–20% of inpatient specimens were positive for NTCD (26, 27). These previous studies cultured stool and plated it on *C. difficile*-specific agar and then defined NTCD as confirmed *C. difficile* without the presence of toxin genes (*tcdA* or *tcdB*). This newly developed NTCD qPCR should improve the efficiency to qualitatively and quantitatively identify NTCD strains, thus facilitating public health and infection control efforts.

This study has certain limitations. Clinical samples were stored at −20°C in the medical microbiology lab and were received at our central laboratory 3–4 days after they were collected from patients. This may have affected the integrity of stool samples and, thus, our results. In our results, we observed only one of 2 NTCD strains was amplified when tested with Lok3/lok1 primer, a highly conserved region in NTCD. This may be due to PCR optimization and will need repeat controlled experiments. Different PCR results were observed between samples that were enriched prior to testing and those without enrichment steps. This may be due to a lack of germination of spores prior to testing. This is a stand-alone assay so separate assays need to be done for NTCD and toxigenic *C. difficile* identification. We will attempt to multiplex the assay in future work. Our spiking studies demonstrated the effectiveness of the assay to detect NTCD as a concentration of 1 × 10^4^ CFU/mL. Epidemiologic studies will be needed to determine the quantity of NTCD needed in the gut microbiome to confer protective effects against CDI. We used one qPCR machine (QuantStudio 5); results will need to be confirmed with other instruments. Lastly, our results demonstrated the analytical sensitivity of this qPCR assay but should not be interpreted as clinical sensitivity.

In conclusion, this study developed and validated a qPCR assay that can uniquely identify non-toxigenic *C. difficile* strains. This innovative assay may be further used for public health, infection control, diagnostic, and treatment approaches to CDI.

## SUPPLEMENTAL MATERIAL

Supplemental material spectrum.01608-24-s0001.pdf Sanger sequencing.

ASM does not own the copyrights to Supplemental Material that may be linked to, or accessed through, an article. The authors have granted ASM a non-exclusive, world-wide license to publish the Supplemental Material files. Please contact the corresponding author directly for reuse.
