# Supplementary material for: A quantitative PCR to detect non-toxigenic Clostridioides difficile
Source: Microbiol Spectr. 2024 Dec 11;13(1):e01608-24. doi: 10.1128/spectrum.01608-24 (PMC11705841; doi:10.1128/spectrum.01608-24)
Supplement: Supplemental material — Sanger sequencing. [file spectrum.01608-24-s0001.pdf]

Supplementary Data
Sanger sequencing of NTCD locus

Sequence 1: Lok3/Lok1 region
PCR with primers set Lok3/Lok1, strain ATCC 43953, sequencing primer Lok1.
Covered region: *cdu1* and *cdd1*
Sequence_Text file:

```
>AWWWAWAYYTGAACMTTCTGGTCTATATGTTGTCCACGCATTAAACATTCCTTTTCATAC
ATTTGTGCTGGGTTTGCATCTTTAAGTGATTTAATAGTTGTATATCCTAACATGATTAAA
GATTTTTCTGTTGCTTTCCCTACCCCTGGAATTTTTTGTAGTTCTCCCATACTTCGCCTT
CTTTCTGGTACATTATTTTTGTATTATACTTAGTACCAGATATTTTTTTATTATAGTTAA
TATTTAATTTTTAATATTTAATTTTTTATTATATCAATTTATTTATGCTTTTTCATCTAT
GTATATTTTACCACCTCTAAAGTACTGAGTCACTTAATTACATCATAATATAGTTTTATA
CAAATAAAATGATATATTTATTTTTTACTCTGCTAATATCAAATTATTTTAAATTAAATT
TAAACATTTAAAAAGAGTTTTATAATTGTAGATAAACTATTACAAAACTCTTTTTAAACA
ACAAATCATATTATATATAAKWRTTCTTCCCAATTTACCACCCAGTTTTCTAACGACTTT
ATCTTTTCCTCTGTTAGCTCCTCTTCGTGAAATGATACTAATAAATTCCTTATAAATTAT
TCTTCAAAAGATTTTGCAMATTTCTTGAWATAACTYWAAATATGCCTTTTMSTTTGAA
```

NCBI BLAST Search/Hit
Query: None Query ID: lcl|Query_8061165 Length: 658

>Clostridium difficile PaLoc insertion site between cdu1 and cdd3, strain HCD52
Sequence ID: HG002392.1 Length: 3864
Range 1: 431 to 1057

Score:1024 bits(554), Expect:0.0,
Identities:609/636(96%), Gaps:11/636(1%), Strand: Plus/Minus

Alignment:

```
Query  17    TCTGGTCTATATGTTGTCCACGCATTAAACATTCCTTTTCATACATTTGTGCTGGGTTTG  76
             ||| |||||||||||||| | |||||||||||||||||||||||||||||||||||||||
Sbjct  1057  TCT-GTCTATATGTTGTCTATGCATTAAACATTCCTTTTCATACATTTGTGCTGGGTTTG  999

Query  77    CATCTTTAAGTGATTTAATAGTTGTATATCCTAACATGATTAAAGATTTTTCTGTTGCTT  136
             | ||||||||||||||||||||||||||||||||||||||||||||||||||||||||||
Sbjct  998   CGTCTTTAAGTGATTTAATAGTTGTATATCCTAACATGATTAAAGATTTTTCTGTTGCTT  939

Query  137   TCCCTACCCCTGGAATTTTTTGTAGTTCTCCCATACTTCGCCTTCTTTCTGGTACattat  196
             |||||||||||||||||||||||||||||||||||||||||||||||||||||| |||||
Sbjct  938   TCCCTACCCCTGGAATTTTTTGTAGTTCTCCCATACTTCGCCTTCTTTCTGGTATATTAT  879

Query  197   ttttgtattatacttagtaccagatatttttttattatagttaatatttaatttttaata  256
             ||||||||||||||||||||||||||||||||||||||||||||||||||||||||||| 
Sbjct  878   TTTTGTATTATACTTAGTACCAGATATTTTTTTATTATAGTTAATATTTAATTTTTAAT-  820

Query  257   tttaatttttattatatcaatttatttatgctttttcatctatgtatattttaCCACCT  316
                   ||||||||||||||||||||||||||||||||||||||||||||||||||||||
Sbjct  819   ------TTTTTATTATATCAATTTATTTATGCTTTTTCATCTATGTATATTTTACCACCT  766

Query  317   CTAAAGTACTGAGTCACTTAATTACATCATAATATAGTTTTATACAAATAAAATGAtata  376
             ||||||||||||||||||||||||||||||||||||||||||||||||||||||||||||
Sbjct  765   CTAAAGTACTGAGTCACTTAATTACATCATAATATAGTTTTATACAAATAAAATGATATA  706
```

```
Query  377   tttatttttactctgctaatatcaaattattttaaattaaatttaaacatttaaaaaGA  436
             ||||||||||| |||||||||| ||||||||||||||||||||||| |||||||||||||
Sbjct  705   TTTATTTTTATTCTGCTAATAGCAAATTATTTTAAATTAAATTACACATTTAAAAAGA  646


Query  437   GTTTTATAATTGTAGATAAACTATTACAAAACTCTTTTTAAACAACAAATCATATTATAT  496
             |||||| |||||||||||||| |||||||||||||||||||||||||||||||||||||||
Sbjct  645   GTTTTACAATTGTAGATAAACCATTACAAAACTCTTTTTAAACAACAAATCATATTATAT  586

Query  497   ATAAKWRTTCTTCCCAATTTACCACCCAGTTTTCTAACGACTTTATCTTTTCCTCTGTTA  556
             ||||   |||||||||||||||||||||||||||||||||||||||||||||||||||||
Sbjct  585   ATAAGC-TTCTTCCCAATTTACCACCCAGTTTTCTAACGACTTTATCTTTTCCTCTGTTA  527

Query  557   GCTCCTCTTCGTGAAATGATACTAATAAATTCCTTATAAA-TTATTCTTCAAAAGATTTT  615
             |||||||||||||||||||||||||||||||||||||||| |||||||||||||||||||
Sbjct  526   GCTCCTCTTCGTGAAATGATACTAATAAATTCCTTATAAAATTATTCTTCAAAAGATTTT  467

Query  616   GCAMATTTCTTGAWATAA-CTYWAAATATGCCTTTT  650
             ||| |||||| || |||| ||  |||||||||||||
Sbjct  466   GCACATTTCTCGATATAAACTCTAAATATGCCTTTT  431
```

Sequence 2:KB1/KB2 region

PCR with primer set KB1/KB1, strain ATCC 43953, and sequencing primer KB2.

Covered region: *cdu1* and *cdd1*

Sequence_Text file:

```
>CTTARTRATTTATAGTTGTATATCCTAACATGATTAAAGATTTTTCTGTTGCTTTCCCTA
CCCCTGGAATTTTTTGTAGTTCTCCCATACTTCGCCTTCTTTCTGGACATTATTTTTGTA
TTATACTTAGTACCAGATATTTTTTTATTATAGTTAATATTTAATTTTTAATATTTAATT
TTTTATTATATCAATTTATTTATGCTTTTTCATCTATGTATATTTTACCACCTCTAAAGT
ACTGAGTCACTTAATTACATCATAATATAGTTTTATACAAATAAAAATGATATATTTWTT
TTTTACTCTGCTAATATCAAATTATTTTAAATTAAATTTAAACATTTAAAAAGAGTTTTA
TAATTGTAGATAAACTATTACAAAACTCTTTTTAAACAACAAATCATATTATATATAAGC
TTCTTCCCAATTTACCACCCAGTTTTCTAACGACTTTATCTTTTCCTCTGTACYYYYCCY
YCYCYCCACCCSGARATCTCACGATTATTAAGGGGGGGATTTCTTAARAYGGRMGRGRKS
RMMCAYMRYMTTWTSSTA
```

NCBI BLAST Search/Hit

Query: None Query ID: lcl|Query_5169005 Length: 558

>Clostridium difficile PaLoc insertion site between cdu1 and cdd3, strain HCD52
Sequence ID: HG002392.1 Length: 3864
Range 1: 529 to 986

Score:760 bits(411), Expect:0.0,
Identities:449/466(96%), Gaps:10/466(2%), Strand: Plus/Minus

```
Query  8    ATTT-ATAGTTGTATATCCTAACATGATTAAAGATTTTTCTGTTGCTTTCCCTACCCCTG  66
            |||| |||||||||||||||||||||||||||||||||||||||||||||||||||||||
Sbjct  986  ATTTAATAGTTGTATATCCTAACATGATTAAAGATTTTTCTGTTGCTTTCCCTACCCCTG  927

Query  67   GAATTTTTTGTAGTTCTCCCATACTTCGCCTTCTTTCTGG-ACattatttttgtattata  125
            |||||||||||||||||||||||||||||||||||||||| | |||||||||||||||||
Sbjct  926  GAATTTTTTGTAGTTCTCCCATACTTCGCCTTCTTTCTGGTATATTATTTTTGTATTATA  867
```

```
Query  126  cttagtaccagatatttttttattatagttaatatttaatttttaatatttaatttttta  185
            |||||||||||||||||||||||||||||||||||||||||||||||       ||||||
Sbjct  866  CTTAGTACCAGATATTTTTTTATTATAGTTAATATTTAATTTTTAAT-------TTTTTA  814

Query  186  ttatatcaatttatttatgctttttcatctatgtatattttaCCACCTCTAAAGTACTGA  245
            ||||||||||||||||||||||||||||||||||||||||||||||||||||||||||||
Sbjct  813  TTATATCAATTTATTTATGCTTTTTCATCTATGTATATTTTACCACCTCTAAAGTACTGA  754

Query  246  GTCACTTAATTACATCATAATATAGTTTTATACaaataaaaatgatatatttwtttttta  305
            ||||||||||||||||||||||||||||||||||||||||| |||||||||| |||||||
Sbjct  753  GTCACTTAATTACATCATAATATAGTTTTATACAAATAAAA-TGATATATTTATTTTTTA  695

Query  306  ctctgctaatatcaaattattttaaattaaatttaaacatttaaaaaGAGTTTTATAATT  365
             |||||||||| ||||||||||||||||||||||| ||||||||||||||||||| ||||
Sbjct  694  TTCTGCTAATAGCAAATTATTTTAAATTAAATTTACACATTTAAAAAGAGTTTTACAATT  635

Query  366  GTAGATAAACTATTACAAAACTCTTTTTAAACAACAAATCATATTATATATAAGCTTCTT  425
            |||||||||| |||||||||||||||||||||||||||||||||||||||||||||||||
Sbjct  634  GTAGATAAACCATTACAAAACTCTTTTTAAACAACAAATCATATTATATATAAGCTTCTT  575

Query  426  CCCAATTTACCACCCAGTTTTCTAACGACTTTATCTTTTCCTCTGT  471
            ||||||||||||||||||||||||||||||||||||||||||||||
Sbjct  574  CCCAATTTACCACCCAGTTTTCTAACGACTTTATCTTTTCCTCTGT  529
```
